# Phylodynamics of major CRF01_AE epidemic clusters circulating in mainland of China

**DOI:** 10.1038/s41598-017-06573-6

**Published:** 2017-07-24

**Authors:** Xiaolin Wang, Xiang He, Ping Zhong, Yongjian Liu, Tao Gui, Dijing Jia, Hanping Li, Jianjun Wu, Jin Yan, Dianmin Kang, Yang Han, Taisheng Li, Rongge Yang, Xiaoxu Han, Lin Chen, Jin Zhao, Hui Xing, Shu Liang, Jianmei He, Yansheng Yan, Yile Xue, Jiafeng Zhang, Xun Zhuang, Shujia Liang, Zuoyi Bao, Tianyi Li, Daomin Zhuang, Siyang Liu, Jingwan Han, Lei Jia, Jingyun Li, Lin Li

**Affiliations:** 1 Department of AIDS Research, State Key Laboratory of Pathogen and Biosecurity, Beijing Institute of Microbiology and Epidemiology, Beijing, 100071 China; 2 Guangdong Provincial Institute of Public Health, Guangdong Provincial Center for Disease Control and Prevention, Guangzhou, Guangdong 511430 China; 3 Shanghai Municipal Center for Diseases Control and Prevention, Shanghai, 200336 China; 4 The 59th hospital of PLA, Kaiyuan, Yunnan 661600 China; 5 Anhui Medical University, Hefei, Anhui 230032 China; 6 Anhui Provincial Center for Disease Control and Prevention, Hefei, Anhui 230601 China; 7 Guangdong Provincial Center for Disease Control and Prevention, Guangzhou, Guangdong 511430 China; 8 Institute of AIDS Control and Prevention, Shandong Center for Disease Control and Prevention, Jinan, Shandong 250014 China; 9 Department of Infectious Diseases, Peking Union Medical College Hospital, Peking Union Medical College and Chinese Academy of Medical Sciences, Beijing, 100032 China; 10 Research Group of HIV Molecular Epidemiology and Virology, Center for Molecular Virology, The State Key Laboratory of Virology, Wuhan Institute of Virology, Chinese Academy of Sciences, Wuhan, Hubei 430071 China; 11 Key Laboratory of AIDS Immunology of National Health and Family Planning Commission, Department of Laboratory Medicine, The First Affiliated Hospital of China Medical University, Shenyang, Liaoning 110001 China; 12 Shenzhen Municipal Center for Disease Control and Prevention, Shenzhen, Guangdong 518055 China; 13 State Key Laboratory of Infectious Disease Prevention and Control, National Center for AIDS/STD Control and Prevention (NCAIDS), Collaborative Innovation Center for Diagnosis and Treatment of Infectious Diseases, Chinese Center for Disease Control and Prevention, Beijing, 102206 China; 14 Sichuan provincial center for disease control and prevention, Chengdu, Sichuan 610041 China; 15 Hunan Provincial Center for Disease Control and Prevention, Changsha, Hunan 410005 China; 16 Fujian Provincial Center for Disease Control and Prevention, Fuzhou, Fujian 350001 China; 17 Zhejiang Provincial Center for Disease Control and Prevention, Hangzhou, Zhejiang 310051 China; 18 School of Public Health, Nantong University, Nantong, Jiangsu 226019 China; 19 Guangxi Center for Disease Prevention and Control, Nanning, Guangxi 530028 China

## Abstract

As the most dominant HIV-1 strain in China, CRF01_AE needs to have its evolutionary and demographic history documented. In this study, we provide phylogenetic analysis of all CRF01_AE *pol* sequences identified in mainland China. CRF01_AE sequences were collected from the Los Alamos HIV Sequence Database and the local Chinese provincial centers of disease control and prevention. Phylogenetic trees were constructed to identify major epidemic clusters. Bayesian coalescent-based method was used to reconstruct the time scale and demographic history. There were 2965 CRF01_AE sequences from 24 Chinese provinces that were collected, and 5 major epidemic clusters containing 85% of the total CRF01_AE sequences were identified. Every cluster contains sequences from more than 10 provinces with 1 or 2 dominant transmission routes. One cluster arose in the 1990s and 4 clusters arose in the 2000s. Cluster I is in the decline stage, while the other clusters are in the stable stage. Obvious lineage can be observed among sequences from the same transmission route but not the same area. Two large clusters in high-level prevalence were found in MSM (Men who have sex with men), which highlighted that more emphasis should be placed on MSM for HIV control in mainland China.

## Introduction

The first case of an HIV-positive patient was reported in 1985 in China^1^. Since then, the number of reported HIV/AIDS cases has increased every year. By the end of 2011, the number of people living with HIV in China was estimated to be 0.78 (0.62–0.94) million^2^ and the HIV epidemic has become a large public health problem in China. Meanwhile, the main population conferred to HIV prevalence in China has also shifted over the last 30 years. The first outbreak of HIV-1 infections in China was identified in 1989 in intravenous drug users (IDUs) in the southwestern provinces^3–5^. In the mid-1990s, a comprehensive HIV outbreak was identified in former plasma donors (FPDs)^6, 7^. Ten years later, the HIV epidemic in China expanded beyond IDUs into men who have sex with men (MSM) and heterosexuals^8, 9^. Since then, the sexual transmission of HIV has expanded quickly; in 2014, sexual transmission accounted for 91.5% of new HIV/AIDS cases^10^. China is now facing a new challenge to curb the rapid spread of the HIV-1 epidemic through sexual transmission^11^.

With the shift of the dominant transmission route in China, HIV subtypes in China have also changed. Subtype B strains were dominant during the early phase of the HIV-1 epidemic in China^12–14^. Then, in the early 1990s, subtype C was introduced into China from India and it became dominant^4, 14^. Soon after that, the co-circulation of subtype B and C led to the generation of CRF07_BC and CRF08_BC, which subsequently rapidly spread in China along the drug trafficking route^15^. At the same time, the Thai B variant was introduced into former blood donors, which lead to the outbreak of HIV-1 in the central provinces of China^7^. After 2000, CRF01_AE quickly spread following the increase of HIV-1 infections due to sexual transmission. In 2006, the overall proportion of CRF01_AE in China reached 27.6%^16^; the proportions were even higher in heterosexual transmitted populations and MSMs (39.8% and 55.8%, respectively)^16^. CRF01_AE was the most dominant strain in most of the areas in China (except some southwestern areas)^16^. CRF01_AE was even responsible for more than 80% of new HIV infections in some provinces^17^. Therefore, it is imperative to understand how the CRF01_AE epidemic is spreading in China.

Due to the stigma against sexually transmitted diseases in China, it is difficult to monitor the HIV epidemic by conventional descriptive epidemiological methods. Recent advances in computational science have allowed us to infer the evolutionary dynamics of a pathogen population from large-scale sequence data using phylodynamics methods^12^, which have been extensively used in the analysis of repaid evolutionary pathogens, including influenza A, hepatitis C, and HIV^1, 4, 6, 9, 13, 18–21^. Since 2008, our laboratory have been collecting HIV-1 nucleotide sequence data from comprehensive areas in China as part of our nationwide surveillance project on HIV drug resistance. In this study, we report our results from applying the phylodynamics approach to these sequence data to understand the trends in the CRF01_AE outbreak in China, genetic relationships between the strains circulating in China, details of their transmission risk factors, and finally, to identify the target populations for effective action plans to prevent further transmission of CRF01_AE in China.

## Results

### Demographic characteristics of CRF01_AE-infected individuals

**Figure 1:**
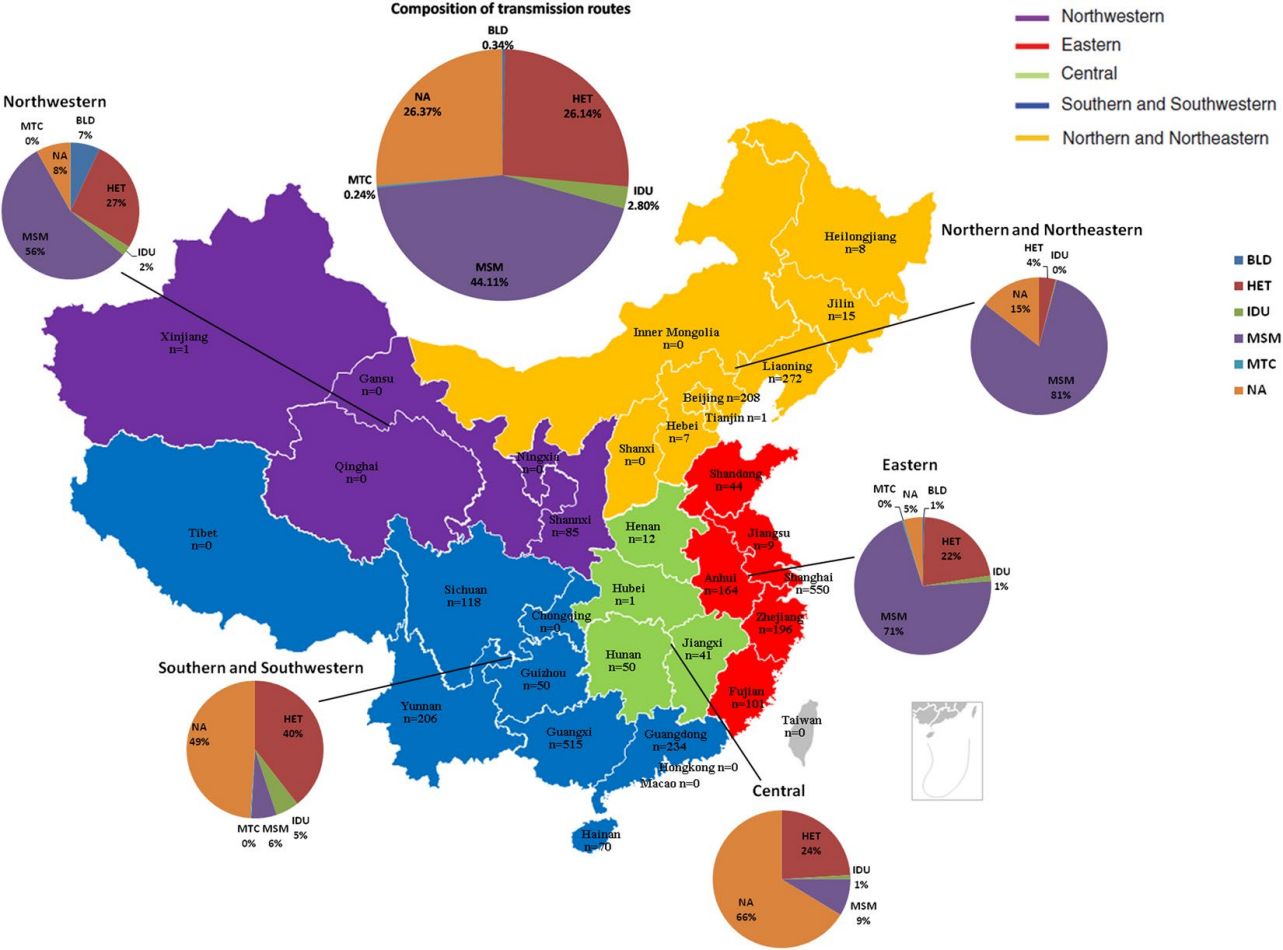
Geographical and transmission route compositions of CRF01_AE sequences enrolled in the study. Map of China with prefecture names and the number of sequences listed on the left side. Transmission routes of sequences were collected and are depicted as a pie chart. Maps were generated with R software 3.3.1 (https://www.r-project.org/).

A total of 2965 HIV-1 subtype CRF01_AE *pol* sequences isolated from mainland China were used to establish the dataset. Of them, 69.7% (2067) of the sequences were retrieved from the Los Alamos HIV Sequence Database and the other 30.3% (898) of the sequences were collected from local centers of disease control and prevention in China. There were 77.4% of the provinces (24) in China that were covered. The areas containing sequences are depicted in Fig. 1. The distribution of transmission routes was also investigated. Of the sequences retrieved from the Los Alamos HIV Sequence Database, 68.0% (1405/2067) contain information on risk factors. The dominant transmission route is men who have sex with men (MSM; 44.11%), followed by heterosexual contact (26.14%), intravenous drug use (IDU; 2.80%), blood borne transmission (0.34%), and mother-to-child transmission (0.24%) (Fig. 1). The sampling years, which were from 1996 to 2014, were obtained from 2883/2965 (97.2%) of the sequences.

### Multiple epidemic clusters were identified

**Figure 2:**
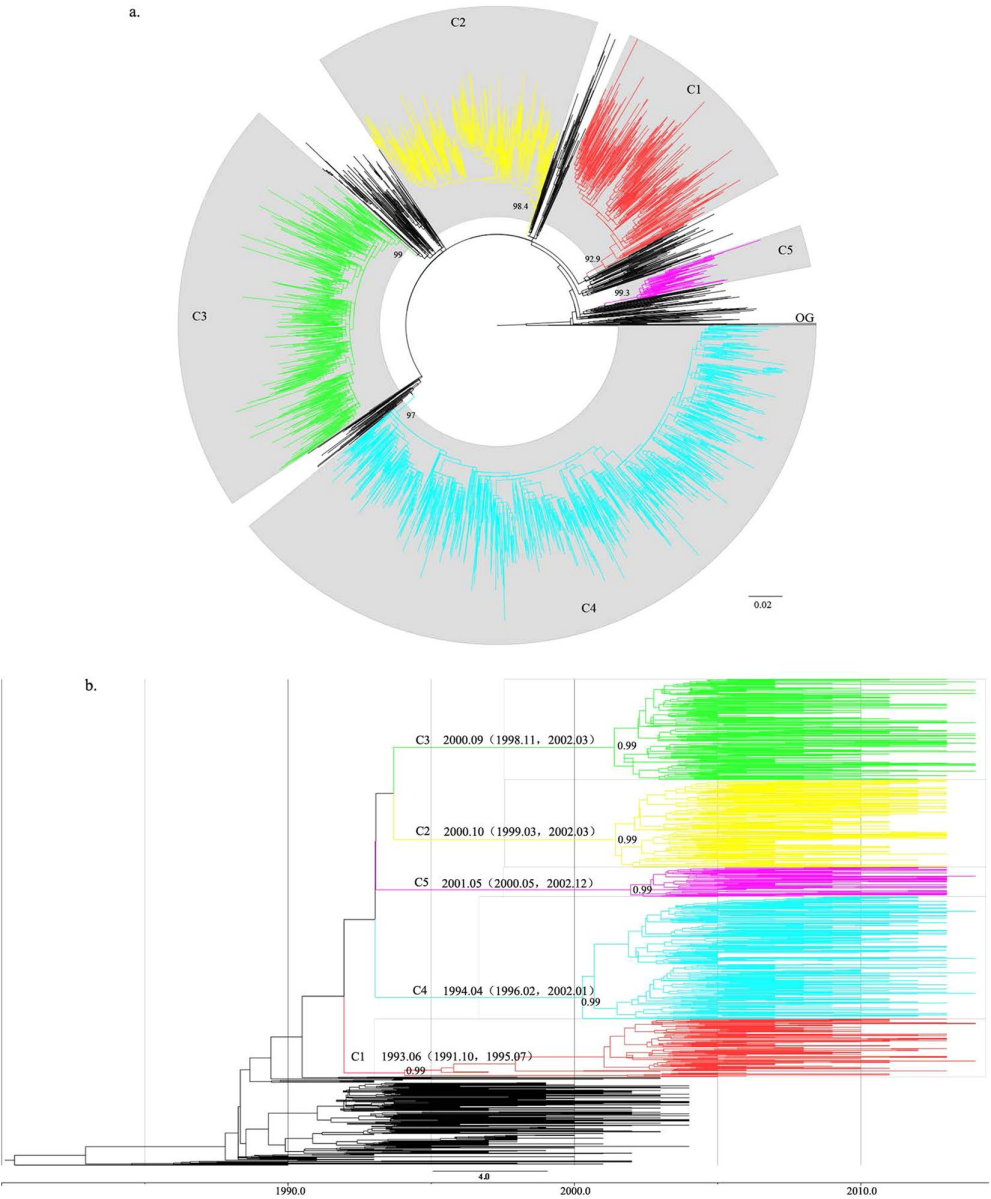
Epidemic clusters labeled in ML tree and MCC tree. (**a**) Maximum likelihood phylogenetic analysis of CRF01_AE *pol* sequences from mainland China. The dataset included 2965 CRF01_AE sequences from 24 Chinese provinces. The tree was rooted using 3 subtype A1 sequences as the outgroup. Five significantly supported monophyletic clusters (numbers inside the monophyletic clades correspond to approximate likelihood ratio test SH-like values) were identified and are colored differently. Branches are scaled in nucleotide substitutions per site according to the bar at the bottom of the figure. (**b**) Maximum clade credibility trees of the CRF01_AE sequences from China based on the partial *pol* gene. There were 939 sequences that were finally selected in the dataset. All of the sequences are labeled in color as in the ML tree. The data of MRCA and posterior probability are labeled next to each node.

**Figure 3:**
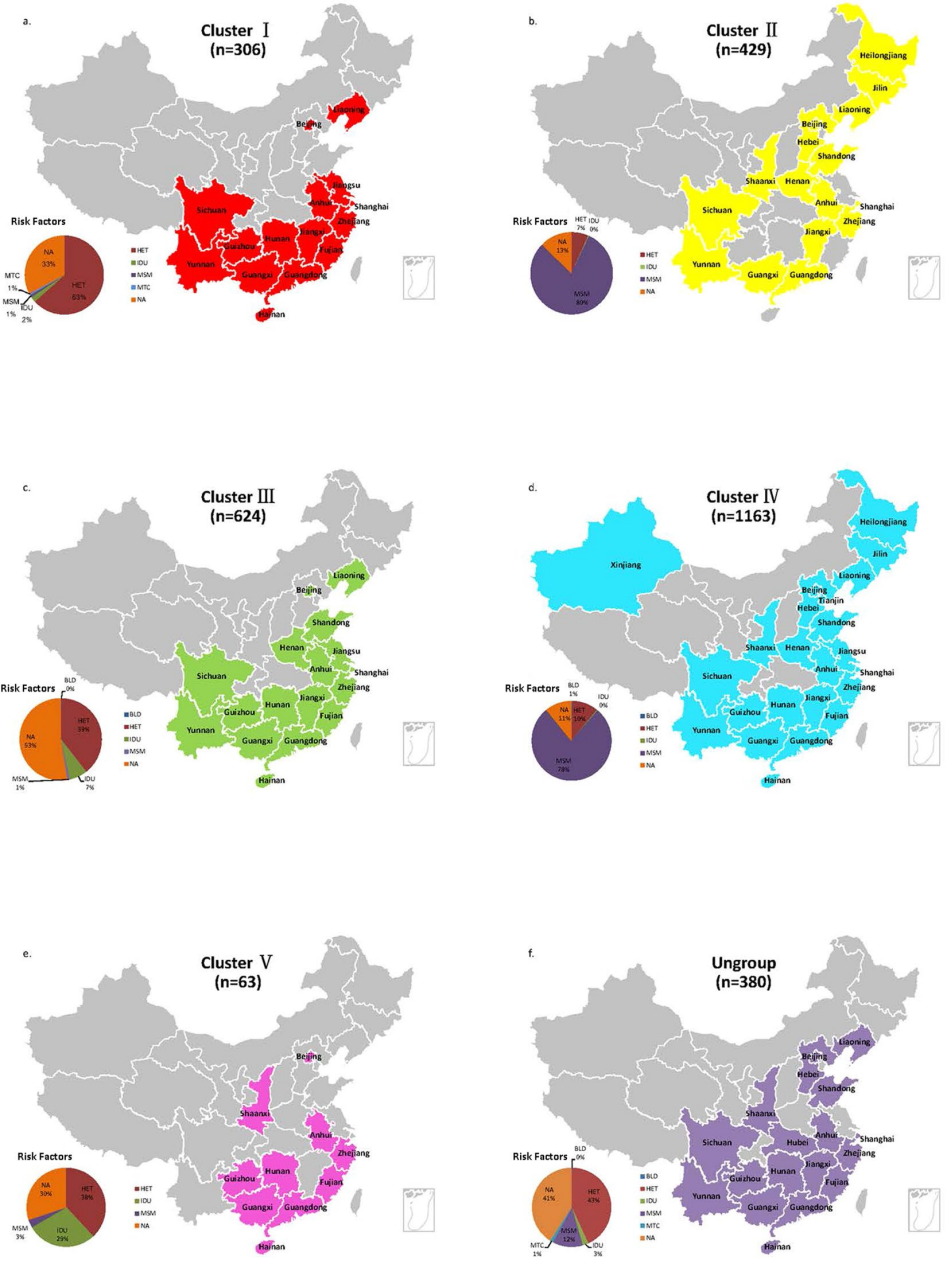
Distribution of Chinese CRF01_AE sequences from different clusters. The number of sequences from different clusters is listed below the cluster name. The provinces containing sequences from different clusters are colored as in the ML and MCC trees. The transmission routes of sequences from each cluster are also depicted below. Maps were generated with R software 3.3.1 (https://www.r-project.org/).

In the ML tree, 5 epidemic clusters (Clusters I to V) were identified (Fig. 2). There were 2585 sequences (87.1% of the total sequences) that were included in those 5 epidemic clusters. The areas of distribution of the 5 major epidemic clusters were comprehensive, and all of the clusters were comprised of sequences from more than 10 provinces (Fig. 3). The dominant transmission routes of the 5 major epidemic clusters were totally different (Fig. 3). Clusters I and III were mainly comprised of sequences from patients infected through heterosexual contact (63% and 39%, respectively); Clusters II and IV were mainly comprised of sequences from MSM (80% and 78%, respectively); and Cluster V was mainly comprised of sequences from IDUs (29%) and heterosexuals (38%). Ungrouped sequences were mainly from heterosexuals (43%) and MSM (12%). The sampling times of sequences from those clusters were 1997–2014, 2003–2013, 2003–2014, 2005–2014, and 2005–2014, respectively.

### tMRCA and evolutionary rate of major epidemic clusters

**Figure 4:**
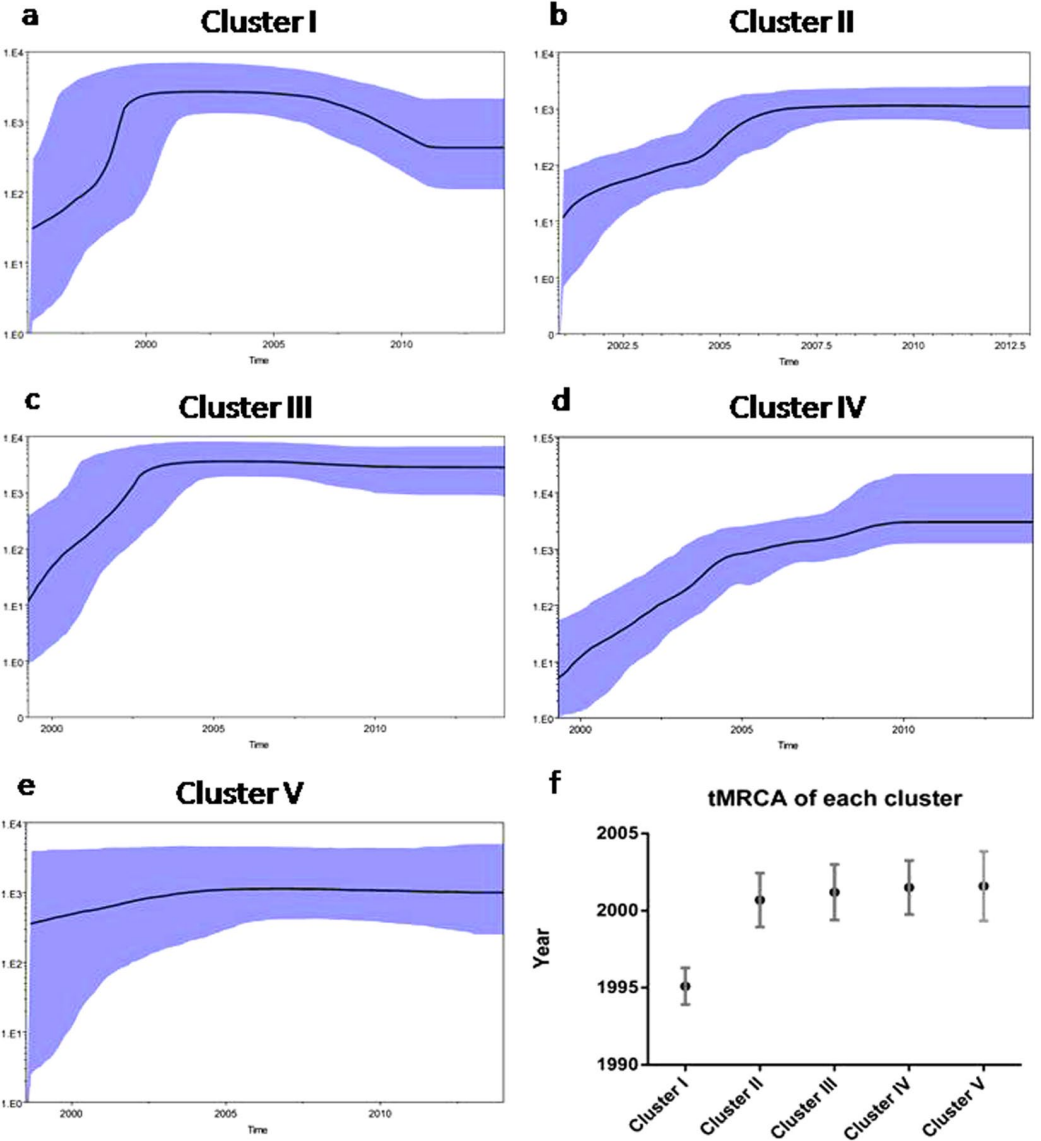
Demographic history of major CRF01_AE epidemic clusters in mainland China. (**a**–**e**) Median estimates of the effective number of infections using Bayesian skyline (solid line) are shown in each graphic together with 95% highest probability density intervals of the Bayesian skyline estimates (blue area). The vertical axes represent the estimated effective number of infections on a logarithmic scale. Time scale is in calendar years. (**f**) tMRCA and 95 CI of each cluster are listed.

A total of 939 sequences were selected to calculate the evolutionary rate and time of most recent common ancestor (tMRCA) of major epidemic clusters. The estimated median evolutionary rates for Cluster I to V are 3.15 × 10^−3^(2.42 × 10^−3^–3.95 × 10^−3^), 3.06 × 10^−3^(2.51 × 10^−3^–3.62 × 10^−3^), 2.54 × 10^−3^(1.94 × 10^−3^–3.15 × 10^−3^), 2.82 × 10^−3^(2.24 × 10^−3^–3.43 × 10^−3^), and 0.95 × 10^−3^(0.35 × 10^−3^–1.49 × 10^−3^) nucleotide substitutions/site/year (95% highest probability density overlap). With these substitution rates, the estimated tMRCAs of the 5 epidemic clusters ranged from 1995 to 2001. Interestingly, only Cluster I was estimated to have been introduced into China in early 1990s, the 4 other clusters were estimated to have been introduced into China around 2000 (Fig. 4f).

### Demographic history of major epidemic clusters

To explore the population growth patterns of major epidemic clusters of CRF01_AE in mainland China, we completed Bayesian skyline plot (BSP) analysis. Different population growth patterns were observed among 5 major CRF01_AE epidemic clusters. Cluster I is characterized by an initial rapid growth period of about 5 years, it reached a stable stage in 2000, and then it declined after 2006. Clusters II and III had an initial rapid growth period of about 5 years and they were in a stable stage until the present. Cluster IV has had a longer growth stage (over 10 years) than the other clusters. Considering that Cluster IV is mainly composed of sequences from MSM, the data suggested that CRF01_AE strains prevalent in MSM may undergo different expanding patterns with other risk populations (Fig. 4).

## Discussion

CRF01_AE is the first identified circulating recombinant form (CRF) of HIV in the world. The earliest CRF01_AE outbreak was found in Thailand in the late 1980s^13, 22, 23^, and it subsequently disseminated into neighboring countries, including Vietnam, Cambodia, Malaysia, Indonesia, and China^12, 20, 24–30^. In this study, we sought to characterize the CRF01_AE epidemic in China. CRF01_AE sequences from comprehensive areas in China were collected for sophisticated phylogenetic analysis. There were 5 large epidemic clusters that were identified. The times of these epidemic clusters being introduced into China were determined and the growth tendencies of these clusters were also demonstrated. One cluster (Cluster IV) containing sequences from MSM showed a tendency for a longer growth period for the effective population size compared to the other clusters. The results provide new information on CRF01_AE prevalence in China, and even in the whole world.

Considering the severe situation of CRF01_AE infections in China, several studies have been completed for phylogenetic analysis of CRF01_AE in China^12, 20, 30, 31^; however, most of them were confined to specific areas or populations, so it could not be depicted as a whole. Up to now, 2 reports can be found on Chinese CRF01_AE phylogenetic analysis based on sequences from relatively extensive areas. The first study was published in 2013^32^. There were 102 CRF01_AE full-length genomic sequences that were collected from 12 provinces in China; 7 clusters were identified, and 5 of them contained less than 10 sequences. The limited number of sequences makes the results difficult to reflect the real situation of CRF01_AE prevalence in China. In the same year, a second relatively comprehensive phylogenetic study was reported based on CRF01_AE sequences obtained from the Los Alamos HIV Sequence Database^33^. There were 1957 sequences spanning the HIV-1 gag p17 region that were included for analysis. The study mainly analyzed CRF01_AE distribution among different countries, and only 2 large epidemic clusters of CRF01_AE were identified in mainland China. Since a very short sequence (199 base pairs) was used for the analysis, the evolutionary signal might not be enough to depict the CRF01_AE epidemic in China in detail. The current study has obvious advantages over the 2 previous reports. First, sequences from more comprehensive areas were included. In addition to CRF01_AE sequences retrieved from the Los Alamos HIV Sequence Database, 898 sequences from 14 provinces were added to the dataset. Second, relatively long sequences were used for phylogenetic analysis, which could provide more evolutionary signals. The results will meet the need for an illustration of spatiotemporal dynamics of HIV-1 CRF01_AE in mainland China. In a recently published paper, the global disperse pattern of CRF01_AE strain was elucidated^34^; however, only 723 CRF01_AE sequences from China were included and only 2 large clusters were identified in the phylogenetic tree. Hence, the collection of CRF01_AE sequences from China in the current study will help to describe the global dispersal pattern of CRF01_AE.

IDU, heterosexual contact, and MSM are the dominant transmission routes of HIV in China. The distribution of different transmission routes among the clusters identified in this study was obviously unequal. Sequences were more likely to cluster based on the transmission route but not on the areas. A significant separation of the CRF01_AE sequences from different populations was observed. Furthermore, different tMRCAs and growth tendencies of each cluster further proved that each cluster was introduced into China separately. Therefore, more actions should be taken to prevent HIV from spreading among the same populations but not the same areas.

The HIV epidemic in MSM continues to grow in most countries^35^. In some modern countries, like the United States of America and the United Kingdom^36, 37^, MSM is responsible for more than half of the new HIV infections. In China, the proportion of those new HIV infections due to MSM has also increased dramatically in recent years^38^. In this study, we identified 2 large clusters composed of sequences from MSM. One of them (Cluster IV) showed a longer growth period of growth stage compared to the other clusters in the skyline analysis. The results further emphasize the importance of controlling the HIV epidemic in MSM.

It has been estimated that there are 17.82 million MSM in China^18^. Due to social discrimination and the cultural stigma associated with MSM in China, 31.5% of HIV-positive MSM engaged in sex with both men and women within and outside of marriage^19^. This behavior facilitates the transmission of HIV and other STDs from high-risk populations to the general population, and may be contributing to the growing number of HIV-positive women infected through unprotected sex. MSM undoubtedly plays a bridging role in connecting high-risk populations with the general population^39^. The special characteristics of the MSM population make them the subject of the emphasis on HIV prevention and behavioral changes.

To our knowledge, the highest number of CRF01_AE strains from the most comprehensive areas was included in this study compared to previous reports^31, 32, 40–44^. However, CRF01_AE strains were not selected randomly from the whole country, which is a potential weakness of our study. Sample bias could be one of the reasons that caused distinct geographical differences among the clusters. The complex dissemination of the CRF01_AE lineages was interpreted with caution. At the same time, it should be noted that CRF01_AE distribution in the whole country is unequal and it is impossible to include all of the CRF01_AE sequences in one study. The findings on cluster determination, distribution, and phylodynamics tendency may reflect situations of CRF01_AE strain prevalence in mainland China. The sequence data will provide information on HIV vaccine designation in the country and the phylotemperal analysis will help to predict and control the entire HIV epidemic in China.

## Material and Methods

### Ethics Statement

This study was approved by the Ethical Review Board of the Science and Technology Supervisory Committee at the Beijing Institute of Microbiology and Epidemiology. The study was performed in accordance with relevant guidelines and regulations of the committee. All of the sequences submitted by the local centers of disease control and prevention were generated by drug resistance surveillance projects, and written informed consent was signed by all of the subjects. All of the analyses were performed by the technicians blinded to the background information of the specimens.

### Selection of CRF01_AE *pol* sequences

All of the CRF01_AE sequences identified in China were retrieved from the Los Alamos HIV Sequence Database (http://www.hiv.lanl.gov). All of the sequences were manually selected in order to maximize the length and the number of segments for analysis. Segments spanning 1000 bp of the *pol* gene, including the entire protease (PR) and partial reverse transcriptase (RT) regions (nucleotide 2253–3252 by using HXB2 as a calibrator), were finally selected for further analysis. There were 144 sequences less than 1000 bp that were excluded from the alignment to avoid sacrificing nucleic acid substitution information from the long sequences. In patients with multiple sequences, only the earliest one was retained by clicking the “one-sequence/per patient” option in the search interface of the Los Alamos HIV Sequence Database website. Additional CRF01_AE sequences from provinces without deposited sequences were collected from local centers of disease control and prevention, which had been obtained using a previously published protocol^45^. The subtype of each sequence was further determined using the NCBI viral genotyping tool (http://www.ncbi.nlm.nih.gov/projects/genotyping/formpage.cgi), online REGA software, and COMET software^46^.

### Phylogenetic analyses and selection of specific CRF01_AE epidemic clusters

Sequences were aligned using MUSCLE software^47^, which also excluded codons associated with drug resistance, according to the 2010 International AIDS Society (IAS) list of reverse transcriptase and protease inhibitor resistant mutations. The alignment was manually edited with BioEdit v7.2. Maximum Likelihood (ML) tree was constructed with the PhyML program^48^, in which GTR + I + G was selected as the nucleotide substitution model and SPR (Subtree Pruning and Regrafting) was used for tree searching. The branch support was calculated using the approximate likelihood-ratio test. The method infers the interior branches using asymptomatic distribution for significance testing. It is accurate and fast, hence suitable for use with large datasets^49^. The final trees were visualized with Figtree v 1.4.2. Monophyletic clades (approximate likelihood-ratio test, 0.90) containing more than 30 sequences were considered as the major CRF01_AE epidemic clusters in mainland China.

### Evolutionary and demographic reconstructions

To infer the time of the most recent common ancestor (tMRCA) of each epidemic cluster, the Markov chain Monte Carlo (MCMC) approach was used step by step as described previously^50^. First, the dataset was balanced to make it representative and maximize its ‘clock-likeness’. Second, intra-subtype recombinant sequences were detected and removed from the dataset with RDP^51^ software. Third, Path-O-Gen (http://tree.bio.ed.ac.uk/software/pathogen/) software was used to test the existence of the molecular clock signal. Fourth, the most appropriate nucleotide substitution model was selected using jModelTest^52^. Fifth, changes in the effective population size through time were estimated using BEAST v 1.7.5^53^. For estimation of tMRCA, a nonparametric model (Bayesian skyline plot with strict clock model) was initially used to infer the demographic information. Then, several parametric models, including the constant population size, exponential growth, logistic growth, expansion growth, and Bayesian skyline, were compared by the Bayesian factor after assuming either a strict or a relaxed molecular clock. All of the models were run to 1 × 10^8^ generations, sampling every 1000th generation. The first 10% of the output was used as a burn-in. Convergence of the estimates was evaluated with generation vs. log probability plots in Tracer v.1.5 using an effective sample size >200. A maximum clade credibility (MCC) tree was generated from the posterior distribution of the trees with TreeAnnotator v 1.6.1, which was further edited with Figtree v1.4.2^53^.

### Statistical analysis

Statistical analyses were completed with the GraphPad Prism version 2.01 (GraphPad Software, San Diego, California, USA). Comparisons of the cluster distribution in different provinces and risk factors were performed with Pearson chi-square test. All of the statistical analyses were two sides, and P < 0.05 was considered to be statistically significant.
